# The influence of ideological education on students’ mental health during the pandemic: An empirical analysis based on big data and intelligent model

**DOI:** 10.3389/fpsyg.2022.940770

**Published:** 2022-10-12

**Authors:** Lianxiang Liu

**Affiliations:** School of Marxism, Nanjing University of Science and Technology, Nanjing, China

**Keywords:** ideological education, students’ mental health, psychological crisis, emotional threshold, psychological intervention, machine learning

## Abstract

Ideological education is an important part of students’ education. Good ideological education can greatly reduce students’ mental health problems. Based on relevant theories of psychology, this study analyzes how psychological crises can be warned against through continuous observation of emotions. Further, a psychological crisis warning model is built based on education big data, providing innovative observation methods and ideas for warning college students about psychological crises. The core idea of the model is that stress events are the external cause and personality and mood changes are the internal causes. The calculation, based on the evaluation of stress events and personality, can draw on different types of emotions and emotional threshold intensities to judge emotions. At the same time, the evaluation is based on time sequences of mood changes to judge the psychological crises that college students face by the level of risk. Combining psychological knowledge and machine learning methods, this study proposes a psychological crisis warning algorithm based on educational data, which predicts the duration and intensity of emotions by calculating stressful events and emotional attenuation. The simulation results show that the proposed algorithm can reflect the emotional changes of college students when they are subjected to stress events, and the effectiveness of the proposed algorithm is preliminarily verified. We conducted timely psychological intervention for the students who received negative stimuli, and the results show that timely psychological intervention and ideological education support are necessary and helpful.

## Introduction

At present, psychological problems faced by college students such as suicides, crimes, and other malignant incidents are on the rise year by year. The effects of these incidents on the daily management of colleges and universities, the overall healthy development of college students, families, and social stability have brought a very serious negative impact, and psychological crises faced by college students have become the focus of attention from all sectors of society. A psychological crisis is a temporary emergency state of psychological imbalance that may cause serious harm to oneself, others, or the society. The psychological crisis will seriously affect the study and life of college students and then lead to all kinds of malignant events.

During the pandemic, health analysis has been studied increasingly (Gupta et al., 2022; Iwendi et al., 2022). Influenced by the closed isolation policy, many of the students’ psychological health problems are not allowed to be ignored. More and more schools set up a health committee to support the student’s physical health, and also established an information support department. Based on the social data survey of ideological education, this paper studies the impact of supportive interventions on students’ mental healthMost scholars agree that the psychological crises of college students can be prevented and controlled. Colleges and universities mainly adopt two ways to prevent and control psychological crises: one is to train students to exercise healthy psychological qualities through general education courses. The second is to find students’ psychological problems through active reporting, scale measurement, psychological counseling, and other ways, and then take targeted preventive measures to reduce the occurrence of crises. Another way is psychological crisis warning; its purpose is to achieve the early prediction of a psychological crisis. The crucial and difficult point of college students’ psychological crisis warning is to screen out students with mental health problems timely and correctly and to implement different attention and supervision levels. Currently, most colleges and universities in China have not established standardized and systematic psychological crisis warning mechanisms for college students. Most of the research on college students’ psychological crises warning remains in the theoretical stage, lacking operable methods. Moreover, the commonly adopted psychological crisis screening method, “statistical analysis based on the clinical diagnostic scale,” has many problems, such as large measurement error, single index, low efficiency, and poor timeliness. Therefore, it is urgent to construct an operable, scientific, and time-effective psychological crisis warning method for college students to deal with the above problems.

The wide application of social media (such as Wechat, Weibo, etc.) and the development of big data technology provides new ideas to solve the above problems. Currently, social media has become the main way for college students to record their lives, express their views, share and communicate, and reflect the real state of college students. Real, accurate, and timely social media big data samples bring new opportunities for reforming college students’ psychological crisis screening methods. Big data technology can record the daily behavior data of college students on social media to analyze their psychological state and characteristics, and quickly discover individual mental health risks, which is conducive to early detection and intervention of college students’ psychological crises. Based on the in-depth mining of real and real-time social media big data, this study establishes a psychological crisis warning model based on the stress response theory and personality theory of psychology, designs corresponding algorithms, and conducts simulation experiments to provide some reference for applying big data to the challenge of providing advance warning for college students’ psychological crises.

## Research review

From the perspective of technological development, college students’ psychological crisis warning methods can be divided into three categories: traditional warning methods, information system-assisted warning, and big data-assisted warning.

### Traditional warning methods

Early screening of psychological crises generally adopts the “statistical analysis based on the clinical diagnostic scale.” This method mainly determines early warning indicators based on psychological theories, develops and designs the scale, and then students fill in the scale actively or passively, so as to judge the psychological crisis state of students through statistical analysis of data. There are now corresponding scales for different types of crisis states. To measure suicidal ideation, typical scales include the Scale for Suicide Ideation (SSIC) developed by Beck et al. (1979), the Adult Suicidal Ideation designed by Reynolds (1991), the Suicide Probability Scale (SPS) compiled by Questionnaire (ASIQ) by Cull and Gill (1982), etc. For the measurement of depressive symptoms, typical scales include the Symptom Checklist 90, SCL-90 (Beck et al., 1961), the Hamilton Depression Scale (HAMD) (Hamiton, 1960), etc. For the measurement of anxiety symptoms, typical scales include the Hamilton Anxiety Scale (HAMA) (Hamilton, 1959), the Self-Rating Anxiety Scale (SAS) (Zung, 1971), the State-Trait Anxiety InVentory (STAI) (Spielberger et al., 1970), etc. In the early stage of psychological crisis screening, the data of “statistical analysis based on the clinical diagnostic scale” were mostly in the form of a paper questionnaire survey, mostly static and experimental data, which had problems such as large measurement error, single index, low efficiency, and poor timeliness.

### Information system-assisted warning

With the continuous development of computers, the Internet, and other information technologies, the digital intelligent cloud platform has penetrated into all aspects of university management, and the application of information systems for early warning has become a normal psychological crisis management mode in universities. The “Chinese College Students mental Health Assessment System” organized and developed by the Expert Steering Committee of Students’ Mental Health Education in Colleges and universities of the Ministry of Education has been tried out by many colleges and universities, and many colleges and universities have independently developed a mental health survey platform for college students. Using these systems to conduct a psychological census of college students overcomes the defects of traditional paper-and-pencil psychological tests, such as time consumption, effort, low accuracy of statistical results, and information feedback lag. However, due to the orientation of the psychological census as “screening questions,” college students often resist the negative label of “having psychological problems” and will not fill in the information truthfully, leading to inaccurate results of the psychological census. In addition to the special psychological survey system, many colleges and universities also use auxiliary student information management systems, such as setting up online consulting, offline screening channels to supervise the students taking the initiative to improve the psychological management information, integrated by students who, during the school-year, participate in the psychological stress events census data. This avoids the restraints of using the contents of a single type of psychological archive. Compared with the traditional paper-and-pencil psychological test, the accuracy and efficiency of the information system have been improved. Still, most of the existing systems can only objectively record the indicators of students’ mental health status, and the data processing mostly stays at the level of simple statistics, access, backup, query, and so on, and the data has not been fully utilized.

### Big data-assisted warning

With the development of big data technology, researchers began to try to use big data technology for psychological crisis warnings. The large amount of data accumulated in the student information system and the data generated by students on social media provide rich data resources and a broad mining space for psychological crisis warnings. Given the data from college student information systems or mental health assessment systems, researchers began using data mining techniques, such as decision tree, association rules, and neural network, to find and predict the psychological crises.

With the wide application of social media, more and more researchers have begun to explore the potential of psychological crisis warning through analyzing social media big data. For the data released by students on social media, researchers mostly use statistical analysis methods in the field of computer science to analyze.

Big data technology in the application of psychological crisis early warning is able to improve the data sources, analysis workload, and time consumption, but based on the current social media algorithms for predicting psychological crises, more big data were found using statistical analysis methods in the field of computer science. It lacks an effective psychological theory model, and cannot really reveal the nature of the psychological crisis.

### Commentary on the current situation

To sum up, scholars have been committed to the study of psychological crisis warning and have begun using data mining technology to seek more accurate warning methods. However, the current research on the application of big data in psychological crisis warning is still in the exploratory stage. There are still some problems to be solved: First, the currently widely adopted method of “statistical analysis based on the clinical diagnostic scale” collects mostly static and experimental data, which has problems such as large measurement error, single index, low efficiency, and poor timeliness. Second, psychological crisis warning based on social media big data is mostly used for a single type of crisis, and the algorithm mostly adopts the statistical analysis methods in the field of computer science, lacking effective theoretical support, resulting in poor interpretability of the model.

Given the realistic needs of current college students’ psychological crisis warning and the shortcomings of existing studies, this study is based on an in-depth mining of real and real-time social media big data, to find or predict students who may have a psychological crisis. To make the model highly interpretable, this study first establishes a theoretical model of early warning based on psychological stress response and personality theory, then designs an analysis algorithm based on the theoretical model, and carries out simulation experiments on this basis. Compared with the traditional early warning mechanism, the early warning of a psychological crisis based on social media big data has the characteristics of operability and strong effectiveness, which can improve the effectiveness and accuracy of early warning to a certain extent, thus helping to reduce the suicide, crime, and other behaviors of college students caused by the psychological crisis. At the same time, this method can also provide direct support for college students’ psychological crisis management and provide tool examples for regional and national implementation of relevant psychological crisis management, which has important practical significance for psychological crisis management.

## Theory proposed

### Stress response theory

A psychological crisis is a psychological response in which an individual cannot deal with internal and external troubles in ordinary ways. It generally occurs when an individual encounters an unavoidable and intense stress event, believing that the stress event will harm the status and safety of the individual after evaluation. After the failure of all the coping means available to them, the individual will have obvious acute emotional, cognitive, and behavioral dysfunction. The stress events mentioned here, also called life events, are the stimuli that cause stress. There are four main types of stress events: physical, psychological, cultural, and social. Somatic events refer to the events that directly act on the individual body to produce stress, such as high temperature, disease, or body attack. Psychological events refer to stress events caused by psychological conflicts, such as having high expectations for a certain exam result. Cultural events refer to events that cause stress due to changes in lifestyle and religious beliefs, such as studying abroad. Social events refer to the stress caused by social events, such as social unrest and interpersonal tension. As for the types of stress reactions (the results of stress), (Lazarus and Folkman, 1984) divided them into three types: physiological, psychological, and behavioral reactions. Among them, psychological reactions generally include emotional reactions and cognitive reactions; common emotional reactions are anxiety, fear, depression, anger, etc. Common cognitive reactions include paranoia, repeated rumination, denial, and selective forgetting. Physiological response mainly refers to changes in physiological indicators such as blood pressure and respiration caused by stress events. Behavioral responses generally include avoidance, regression, hostility, self-pity, etc.

**FIGURE 1:**
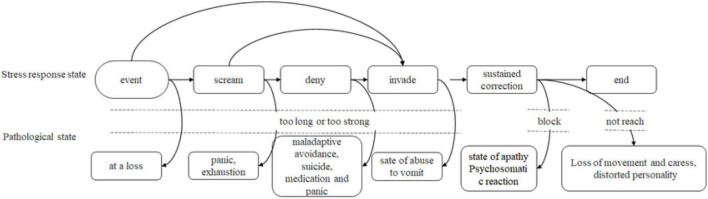
Stress response process model.

The stress response triggered by the stress event involves a certain process. Based on this, Horowitz proposed the stage model of the stress response, as shown in Figure 1. The model divides the stress response process into five stages: scream, denial, invasion, constant correction, and closure. Two stages of denial and invasion are often observed clinically, while the occurrence, degree, and sequence of other stress response states vary from person to person. The denial stage usually has comprehensive symptoms such as emotional numbness, concept avoidance, and behavior constraint. The intrusive stage is the direct, symbolic, conceptual, or emotional repeated reappearance or flashback of the stressful event, such as nightmares about the stressful event, repeated spontaneous images, or surprise reactions derived from other events. When the stress response is too strong or lasts too long, it can cause the individual to develop pathological symptoms, such as panic, exhaustion, and even suicidal thoughts and other crisis states.

Research has shown that stress reactions caused by stress events are mainly psychological reactions, and psychological reactions are mainly reflected in emotional reactions, which not only refer to the negative emotions felt when the stress events cannot be dealt with, but also refer to the positive emotions generated when the stress events can be solved. When people and their environment are not in balance, individuals will enter into a stress state when they believe that the unbalanced state will threaten their own safety and status after evaluation. When individuals lack corresponding social support, a lack of coping skills will produce a lot of emotional problems, such as tension, anxiety, depression, and so on. As individuals cannot bear extreme tension and anxiety, they may experience emotional collapse or want to seek relief, which will lead to emotional imbalance and thus enter a crisis state. Through the above analysis, it can be concluded that emotion is a direct manifestation of psychological crisis. Drastic emotional changes or prolonged negative emotions may indicate that students have entered a state of crisis. Therefore, it is a feasible method to detect psychological crises by identifying and continuously observing those students whose mood changes dramatically and whose negative mood lasts for a long time.

### Personality theory

As for the types of stress reactions (the results of stress), Lazarus and Folkman (1984) divided them into three types: physiological, psychological, and behavioral reactions. Stress events are external triggers for individual emotional responses. However, in the face of the same stress events, each individual has different ways of dealing with them, resulting in various emotional responses, which are largely influenced by individual personality traits. A large number of empirical studies have shown that personality is an important factor affecting emotional expression (Watson and Clark, 1984; Zelenski and Larsen, 1999; Martin et al., 2000; Lucas and Diener, 2001). At the same time, the research also confirmed that psychological crisis and individual personality have a certain relationship. Personality traits are the dynamic organization of the internal mind-body system that determines a person’s behavior and thoughts (Miwa et al., 2001). Different psychologists have put forward other personality models, among which the “Big Five” personality model (Corr and Matthews, 2009) is widely accepted.

The “Big Five” personality model divides people into five types: open, cautious, extroverted, agreeable, and neurotic. People with open personality characteristics are imaginative, emotional, and creative. The cautious personality type has the characteristics of justice, prudence, restraint, and so on; the extroverted personality is characterized by enthusiasm, decisiveness, adventure, and objectivity. The agreeable personality type is associated with confidence, forthright, compliance, and other characteristics; Neurotic personality traits include anxiety, hostility, impulse, and other characteristics. Personality traits are the internal cause of students’ personalized emotional changes. For example, students with open personalities are more optimistic when facing setbacks and will be more active in solving difficulties. Students with neurotic personalities tend to behave negatively in the face of the same frustration.

### Psychological crisis warning model construction

**FIGURE 2:**
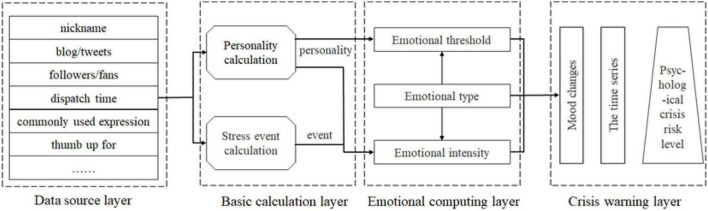
Psychological crisis warning model based on social media big data.

According to the above theoretical analysis, the emotional response is a direct manifestation of a psychological crisis, so that a psychological crisis can be predicted through continuous observation of emotions. The social media big data mentioned in this study mainly refers to the texts and pictures posted by students on social media. Direct emotion observation based on this (such as the text emotion classification method in the computer science field) is often not accurate enough. Personality traits and stressful events are internal and external causes of emotional changes. Therefore, this study proposes to predict emotions based on stressful events and personality and constructs a psychological crisis warning model based on social media big data, as shown in Figure 2. The emotional performance was judged by calculating the emotional intensity and threshold of different types of emotions, and the risk level of the psychological crisis faced by students was judged based on the changes in emotions in the time series. If the emotional intensity is too strong (the emotional change is drastic) or the negative emotional duration is too long, it indicates that the student is at risk of psychological crisis.

## Modeling preparation

According to the student campus online management data set, a campus network is one of the most popular social media apps among college students’ age group. The campus network has accumulated a large amount of user data with objectivity and timeliness, and its data can be obtained through API. Therefore, this study mainly obtains data from this social media. Through the API provided by the campus network, the student data can be collected, processed into a unified data format (JSON or XML, etc.), and stored in the database to provide data support for the basic computing layer. We took a total of 500 samples from the same university and followed the students.

### Basic calculation layer

The basic calculation layer mainly includes two kinds of calculation:

The first one is stress event calculation. Stress event calculation analyzes the texts posted by students on social media and identifies the relevant stress events expressed by students. The scale of LCU (Life Change Units) prepared by Holmes and Rahe (1967) divided stress events into 43 basic types. Chinese scholars combined the LCU scale to study the impact of different life events on Chinese people, and further proposed the SCALE of Life Events Units (LEU), which divides life events into 65 types and gives LEU values of youth, middle age, older age, and old age, respectively. College students are in their youth. Therefore, 15 stress events that college students are more likely to face are selected from the youth group data of the LEU scale in this study, which are as follows: death of parents, divorce of parents, dismissal, serious illness of family members, lovelorn, outstanding achievements and honors, serious illness and trauma, beginning of love, administrative disciplinary action, frustration in entering school, joining the Party and league, dropping out of school, failing in school or employment, and learning difficulties. In this study, the dictionary method was used to calculate stress events. First, according to relevant psychological research, the text expression (words, context) of students in social media corresponded to stress events, so as to construct a dictionary of students’ stress events. Accuracy and efficiency are used to ascend the dictionary, which was first screened by artificial means. In this study, the vocabulary was directly associated with stress events, then a tencent AI lab building word vector model was used to identify and artificially extract words with similar meanings of other words, manually removing text that did not meet requirements, the results are shown in Table 1. In the actual calculation, if the microblog text contains a word in the dictionary, it is considered that the text expresses the corresponding stress event. Taking learning difficulties as an example, the corresponding words and microblogs in the dictionary are shown in Table 2.

**TABLE 1:** Word vector model recognition (section).

Stress events	Artificial vocabulary extraction	Tencent word vector model recommended similar meaning words
Learning difficulties	Learning difficulties	Learning disabilities, attention disorders, school performance decline, **mood disorders**, attention deficits, **behavior problems**, learning problems, learning behind, poor learning ability, dyslexia
Drop out of school	Drop out	Suspension, university withdrawal, expulsion from school, dropout, abandonment, withdrawal from school, transfer, withdrawal
Beginning be in love	Take off a single	Say goodbye to being single, find a partner, **single**, solve the problem of being single, find true love, find a boyfriend, **so be single**, meet true love, find true love, find a partner
Severe trauma	Injured	Injury, major injury, shoulder injury, collision, leg injury, injury condition
Getting into school is a setback	Flunk a competitive examination	After failing, fail, fail, fail in the exam, college entrance examination failure, in the exam, even test, **be admitted**, fail in the exam

**TABLE 2:** Words and examples corresponding to stress events of learning difficulties.

Vocabulary	Examples of microblog content
Learn trapped	Long difficult sentences is really long and difficult, the school is tired π_π, I’ve heard that early to bed and early to rise makes the brain better, try it today, Go for it.
Learning difficulty	Life is hard, It’s hard to study, hard to find a job, hard to find a mate; There is no simple thing.
Study slag	Maybe this life is doomed to be a poor performance of students, daytime five multiple choice questions on the sleepy not……, Let’s study together as a family
Learning disability	A GH course on the sleepy, a brush micro blog is very spiritual. Is it a learning disability
Hopeless students	The last stubborn thing that young people have to do before learning is to buy a lot of stationery.

The second part of the study was a personality calculation. A number of studies have found that personality affects users’ behavior patterns on social media (Ross et al., 2009; Back et al., 2010). Therefore, the personality types of college students can be analyzed through collecting and calculating user data on social media. Through comprehensive analysis of various data of students on social media, students’ personality characteristics can be calculated using the deep learning method. Currently, there have been related studies on personality calculation based on the “Big Five” personality model in Chinese social media. In this study, the Heterogeneous Information Ensemble (HIE) processing framework proposed by Wei et al. (2017) was adopted for personality calculation, which took the “Big Five” personality as the standard. Based on the multidimensional data such as microblog text, user profile picture, facial expression, and interaction form, the semantic information of different features is extracted by the deep learning method, and then the semantic information from other features is integrated based on the stack generalization method, and finally, a prediction result of personality type is formed.

### Emotional computing layer

Based on summaries of previous studies, this study proposed an emotional calculation algorithm based on personality and stress events. The core idea of the algorithm is as follows: First, the emotional intensity values corresponding to students’ various emotional types at a certain moment were obtained based on personality calculation and stress events. Second, the threshold values of positive and negative emotions were estimated according to the students’ personality traits. Finally, whether the emotional intensity of each emotion exceeded the corresponding threshold was judged, so as to judge which emotion the student expressed. In this algorithm, the emotion type adopts the emotion classification method proposed by Ekman and Friesen (1971), which divides emotions into six types: disgust, anger, surprise, fear, happiness, and sadness. Disgust, anger, fear, and sadness are negative emotions, while surprise and happiness are positive emotions.

### Emotional intensity calculation

Emotional intensity refers to people’s tendency toward choices. Combining the results of personality calculation and stress event calculation, the corresponding emotional intensity values of six emotional types of students at a certain time can be calculated. Emotional intensity values are distributed in the range of [−1, 1], in which positive emotions are distributed in the range of [0,1], and negative emotions in the range of [−1, 0).

### Estimation of the emotional threshold

The study of Izard (1977) shows that everyone has an emotional activation threshold, and when the emotional intensity exceeds this threshold, the individual’s emotions can be expressed. For the calculation of the emotional threshold, most of the existing studies set it as a constant value. This study creatively proposed to estimate the threshold value of positive and negative emotional expression according to the personality traits of students, and judge whether the students will express a certain emotion according to whether the emotional intensity exceeds the threshold value. Therefore, the calculation of the intensity of various emotional types is an important basis to judge whether students express such emotions.

### Crisis warning layer

In this study, the risk of the psychological crisis was judged according to the changes in time series through continuous observation of emotions. The research was done mainly through the observation of the following two situations: one is the dramatic change of emotion, that is, from positive emotion to negative emotion or from negative emotion to positive emotion in a short period of time; second, negative emotions last too long. Therefore, this study proposed a dual monitoring method considering the warning value and duration to judge the crisis risk level of students. Combined with continuous-time sequence on all types of the mood change process (such as “anger” being the mood last expressed) and emotional intensity adjacent time change (such as changing from a negative to a positive mood with extreme emotions) these were used to calculate the corresponding warning value in Gupta et al. (2022) intervals, and a system of five early warning signals was visualized, as shown in Table 3, the closer the value is to 1, the higher level of risk. The frequency of college students’ microblog behaviors (such as posting, etc.) will have an impact on the calculation of drastic emotional changes and negative emotional duration. Therefore, in this study, the interval of the time series is set in days. If the learners post more than twice in a day, the maximum emotional value is, respectively, used as the positive and negative emotional value of the day.

**TABLE 3:** Five levels of warning signal system.

Early warning value	Level	Color
[0,0.2]	Security	Green
(0.2,0.4)	Safer	Blue
[0.4,0.6]	Criticality	Yellow
(0.6,0.8)	More dangerous	Orange
[0.8,1]	Dangerous	Red

## Construction of emotion recognition algorithm

**FIGURE 3:**
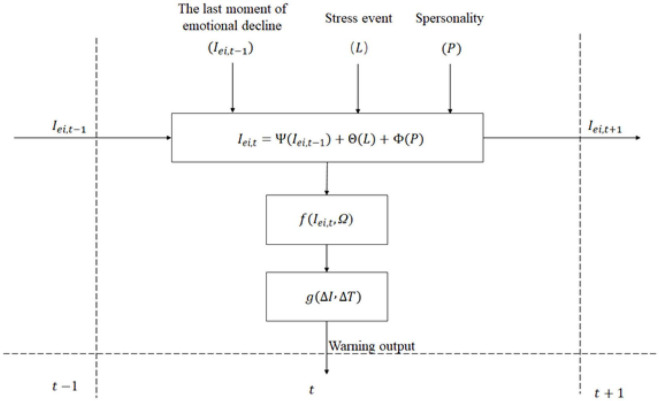
Algorithm structure.

In this study, to avoid the probability problems existing in the pure use of the machine learning algorithm, we combined the knowledge in the field of psychology and the machine learning algorithm to conduct modeling analysis. On the basis of summarizing previous research and emotional psychology theories, the algorithm of emotional prediction is improved and an estimation method of emotional threshold is proposed. The algorithm structure is shown in Figure 3.

Matrix *E* = [*e*_*dis*_, *e*_*ang*_, *e*_*sur*_, *e*_*fea*_, *e*_*joy*_, *e*_*sad*_] is used to represent the emotion type, in which the elements, respectively represent six emotions: disgust, anger, surprise, fear, happiness, and sadness. *L* = [*l*_1_, *l*_2_, ⋯ , *l*_*k*_]” emotions by stress events, respectively. The matrix *P* = [*p*_*o*_, *p*_*c*_, *p*_*a*_, *p*_*n*_] represents the personality types, representing the spatial distribution positions of open, cautious, extroverted, agreeable, and neurotic types, respectively. For example, students with personality space *P* = [0.8, 0.2, 0.7, 0.5, 0] have an open personality and can overcome difficulties with an optimistic attitude when facing difficulties. *I*_*t*_ = [*i*_*e_dis_*,*t*_, *i*_*e_ang_*,*t*_, *i*_*e_sur_*,*t*_, *i*_*e_fan_*,*t*_, *i*_*e_jp_*,*t*_, *i*_*e_sad_*,*t*_] represents the set of emotional intensity at time “t,” indicating the emotional intensity of disgust, anger, surprise, fear, joy, and sadness at time “T”, respectively. Ω = [ω_*pos*_, ω_*neg*_] is the emotional threshold matrix, in which ω_*pos*_ represents the threshold of positive emotion and ω_*neg*_ represents the threshold of negative emotion.

Ψ() represents the attenuation function of emotions. Θ() represents the influence function of stress events. Φ() represents the influence function of personality. f() represents the calculation function of emotions. g() represents the warning function. I_ei,t_ represents the emotion intensity value of the ith emotion at time t, and the attenuation function Φ() is related to the emotion intensity value I_ei,t−1_ of the ith emotion at time t − 1; Stress event influence function Θ() is related to the stimulus L of a stress event suffered by individuals at time t . The personality influence function Φ() represents the influence component of personality factors on emotional intensity. As the individual’s personality is relatively stable, the personality influence function is usually constant. Therefore, this paper assumes that the influence of personality on emotional intensity is a set of constant vector values in a period of time. Emotion calculation function *f*() integrates emotion intensity *I*_*ei,t*_ and emotion threshold at time *t* to determine whether a certain emotion is activated. The psychological crisis warning function *g*(*E*_*i*_, *t*) calculates the psychological crisis level of students according to the calculated emotional intensity and time series values.

### Emotion attenuation function

According to the third law of emotional intensity (Attenuation law of emotional intensity), the attenuation of emotional intensity is actually similar to the exponential function *y* = *e*^−λ^, that is, emotional intensity will decay with time. When at time *t*, the emotional intensity is affected by time t-1, λ is called the attenuation factor, the larger λ is, the faster the emotional intensity decays. Therefore, emotional attenuation function can be defined as Ψ(*I*_*en*,*t*_) = *I*_*en*,*t*−1_*e*^−λ^, in which *n* = {*dis*, *ang*, *sur*, *fea*, *joy*, *sad*}.

### Influence function of stress events

A stress event is the direct factor of emotion change, therefore, Θ(*L*_*k*,*Ie_fea_*,*t*_) = [*L*_*k*_, *I*_*dis*,*t*_, *L*_*k*_, *I*_*eng*,*t*_, *L*_*k*_, *Ie*_*sur*,*t*_, *L*_*k*, *I*e*_fea_*,*t*_, *L*_*k*_, *Ie*_*jop*,*t*_, *L*_*k*_, *I*_*exad*,*t*_], in which *L*_*k,Ie_fea,t_*_ represents the amount of influence of the kTH stress event on the nTH emotion.

### Personality influence function

A person’s personality is relatively stable, so the influence of personality on emotion can be considered as a certain weight in a period of time. Research shows that it is difficult to directly infer the influence of personality on emotion. In order to construct the relationship between personality and emotion, some scholars proposed a “character-motion-emotion-expression” model in 2002 (Kshirsagar, 2002), and then Gebhard put forward the “character-motion-emotion” model (Gebhard, 2005). In this model, PAD three-dimensional mood space was first introduced as an intermediary between personality and mood, and the mood space was measured by three dimensions of Pleasure, Arousal, and Dominance (Mehrabian, 1996). Some Chinese scholars have also explored this aspect by using the algorithms from existing literature (Malekshah and Ansari, 2021; Malekshah et al., 2021). In order to calculate the relationship between personality and mood, we introduce the mapping matrix K and the mood space matrix M . The equation of personal-mood transfer is *M* = *P* × *K^T^*, in which *K* is processed by Gebhard (2005). In order to calculate the relationship between mood and emotion, we introduce the “motion-emotion” transfer matrix *F* and the 24-dimension emotion space matrix *O*. The motion-emotion transfer equation is *O* = *M* × *F*, in which *F* adopts the same treatment method as Gebhard’s study (Gebhard, 2005) .

The above 24 dimensions of basic emotions are mapped to the 6 dimensions of basic emotions proposed by Ekman. The calculation method is as follows.


$$I_{p,dis}=O_{Disgust}$$

$$I_{p,ang}=\left(O_{Anger}+O_{Reproach}+O_{Hate}\right)\times 1/3)$$

$$I_{p,sur}=O_{Surprise}$$

$$I_{p,fea}=\left(O_{Fear}+O_{FearsConfirmed}\right)\times 1/2$$

$$\begin{array}{c} I_{p,joy}=O_{HappyFor}+O_{Gloating}+O_{Joy}+O_{Pride}+O_{Admiration}\\ +O_{Liking}+O_{Love}+O_{Hope}+O_{Satisfaction}+O_{Relief}\\ +O_{Gratification}+O_{Relief}+O_{Gratification}+O_{Gratitude})\times 1/12 \end{array}$$

$$\begin{array}{c} I_{p,sad}=(O_{Resentment}+O_{Pity}+O_{Distress}+O_{Shame}+O_{Remorse})\times 1/5 \end{array}$$

Therefore, the function of personality’s influence on emotion is as follows.


$$\text{Φ}\left(P\right)=\left[I_{p,dis},I_{p,ang},I_{p,sur},I_{p,fea},I_{p,joy},I_{p,sad}\right]$$

### Emotion calculation function

#### Emotion intensity function

The emotion intensity function at time *t* can be expressed as:


$$I_{en,t}=\text{Ψ}\left(I_{en,t-1}\right)+\text{Θ}\left(L\right)+\text{Φ}\left(P\right)$$

That is, the emotional intensity at time *t* is the sum of the attenuation value of the emotional intensity at the previous time (*t* − 1), the influence of stress events on emotions and the influence of personality factors on emotional changes.

#### Threshold estimation function

At present, most studies set the emotional threshold as a constant value, and some researchers define the relationship between emotional threshold and personality as a linear function. However, in real life, different people show different reactions when facing the same stressful events. Therefore, the estimation of emotional threshold with a simple fixed value or linear relationship has great limitations. A new estimation method of emotional threshold was proposed based on the research of Watson and Clark (1984, 1992). Watson and Clark study showed that conscientiousness (C) and extraversion (E) had significant influence on positive emotions. Neuroticism (N) personality has significant influence on negative emotion.

**FIGURE 4:**
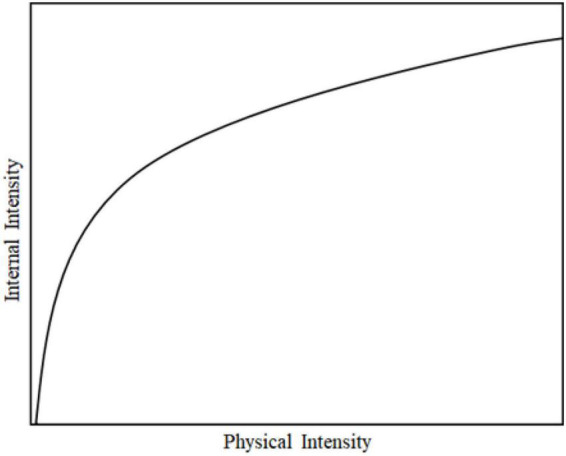
The relationship between the amount of physical stimulus and the subjective internal response.

According to the theory of Izard (1977), the lower the threshold of a certain emotion, the easier it is for a person to show this emotion. For example, when a person’s threshold of sadness is low, a small thing will often trigger his strong sadness. Therefore, this study assumes that the threshold is only related to cautious, extroverted and neurotic personalities. Let ξ indicate the difference between the personality (C,E) affecting positive emotion and the personality (*N*) affecting negative emotion, i.e., ξ = *p*_*c*_ + *p*_*e*_ − *p*_*n*_; Let ω be the emotional threshold, which is closely related to ξ. A larger ξ value indicates that the learner’s positive personality is greater than negative personality, and the threshold of negative emotion is larger. The smaller the threshold of positive emotion is, the easier it is to show positive emotion, and the harder it is to show negative emotion. Kingdom and Prins (2016) mentioned that the psychophysical field constructed a general transition function through a series of experiments to describe the relationship between the amount of physical stimulus and the subjective internal response, as shown in Figure 4. With the increase of stimulus difference, the increase of internal sensation will become slower and slower.

Based on the above theory, this study assumes that there is a similar relationship between the emotional threshold and ξ, which is expressed by function Ω, in which ω_*neg*_ represents the negative emotional threshold and ω_*pos*_ represents the positive emotional threshold. Considering the negative value of ξ of the personality difference, we propose the functionarctan() to characterize, and adding $\frac{\pi }{2}$ and dividing π to the function is to scale the estimation to the interval of [0,1].


$$\Omega =\left\{ \begin{array}{c} \omega_{neg}=\frac{\text{arctan}\left(\xi \right)+\frac{\pi }{2}}{\pi }\\ \omega_{pos}=1-\omega_{neg} \end{array}\right.$$

**FIGURE 5:**
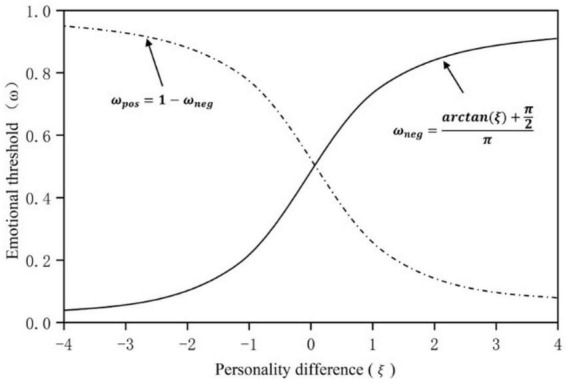
Simulation image of emotional threshold function.

The simulation image of the function of this estimation method is shown in Figure 5. It can be seen that the larger the ξ, the larger the negative emotion threshold is, indicating that the negative emotion is more difficult to be activated and its growth rate is slower and slower.

#### Emotion calculation function

The emotion intensity value derived from the emotion intensity function is compared with the emotion threshold value calculated based on personality. If the emotion exceeds the threshold value, the emotion is considered to be expressed, otherwise it is not expressed. In order to facilitate the calculation, we set the two emotional intensities of happiness and surprise as positive emotions, namely: *I*_*pos*_ = [*i*_*e_joy_*,*t*_, *i*_*e_sur_*,*t*_], and set disgust, grief, fear, and sadness as negative emotions, namely: *I*_*neg*_ = [*i*_*e_dis_*,*t*_, *i*_*e_ang_*,*t*_, *i*_*e_fac_*,*t*_, *i*_*e_sad_*,*t*_]. The emotional calculation equation at time *t* is as follows.


$$f\left(i_{e_{n},t},\text{Ω}\right)=\left\{ \begin{array}{c} i_{e_{n},t},I_{pos}\text{∼}w_{pos}∥I_{neg}\text{∼}w_{neg}\\ 0,I_{pos}<w_{pos}∥I_{neg}<w_{neg} \end{array}\right.$$

### Psychological crisis warning function

According to the theoretical model constructed in this study, it is necessary to make statistics on the changes in the intensity and duration of emotions. If the emotion intensity changes beyond a certain range, it will be considered as a drastic change, which may cause the psychological crisis state of students. For example, the positive emotion with the intensity of 0.8 (*I*_*pos*_ = 0.8)will change to the negative emotion with the intensity of −0.8(*I*_*neg*_ = −0.8). At the same time, if the negative emotion lasts too long, such as more than 3 days, it is easy to lead students into a state of psychological crisis.

Therefore, this study proposes a psychological crisis warning algorithm, which inputs the difference value Δ*I* of six emotions or the time difference value Δ*T* of negative emotions. In the algorithm, Δ*I* = |*I*_*neg*_| − *I*_*pos*_. In this study, the Logistic function is used as the early warning algorithm, as follows.


$$g\left(\text{Δ}I,\text{Δ}T\right)=\frac{1}{1+\text{exp}\left(-\text{Δ}I,-\text{Δ}T\right)}$$

## Simulation experiment

The simulation is the computer simulation method before entering the real experiment. The operating system of the simulation machine used in this study is Windows 10, and the CPU model is I5-11400H. The simulation program is written in Python and the software package is Numpy. In order to conduct simulation experiments, this study randomly set the personality space *P*_*o*_ = [0.8, 0.2, 0.6, 0.4, 0] of open personality and *P*_*n*_ = [0, 0.6, 0.1, 0.1, 0.9] of neurotic personality. Meanwhile, Random set positive stimulus component *w*_*pos*_ = [0.2, 0.1, 0.3, 0.1, 0.7, 0.3], negative stimulus component *Lw*_*eng*_ = [−0.5, −0.7, −0.1, −0.4, −0.5, −0.5]. Furthermore, the emotional changes of open personality and neurotic personality under single and multiple stimuli were simulated, respectively.

### Positive stimulus

**FIGURE 6:**
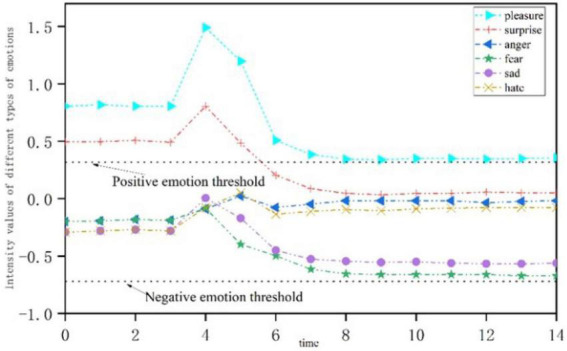
Emotional change process of open personality stimulated by a single positive stress event.

Figure 6 shows the emotional change process of open personality under a single positive stimulus. When the personality is subjected to a positive stimulus at *t* = 3, the intensity of positive emotion increases at first, while the intensity of negative emotion decreases. As time goes by, both positive and negative emotions gradually decline. Among them, positive emotions were activated, showing “pleasure” and “surprise” emotions at first, and “pleasure” emotions were still displayed after attenuation, while “surprise” emotions were no longer displayed, and negative emotions were not activated throughout the whole process.

**FIGURE 7:**
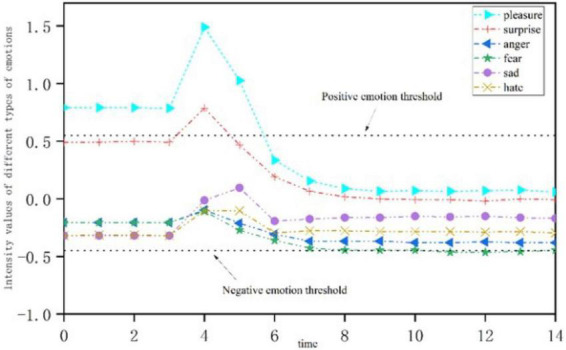
Emotional change process of neurotic personality stimulated by a single positive stress event.

Figure 7 shows the emotional change process of neurotic personality under a single positive stimulus. When the neurotic personality is subjected to a positive stimulus at *t* = 3, the intensity of positive emotion increases at first, while the intensity of negative emotion decreases. As time goes by, both positive and negative emotions gradually decline. First, positive “pleasure” and “surprise” emotions were activated. The intensity of these two positive emotions increased at the initial stage of the stimulus, and then decreased to below the threshold of positive emotions. However, the negative emotion was not activated at the initial stage of the stimulus. As time went by, the emotion of “fear” broke through the negative emotion activation threshold.

### Negative stimulus

**FIGURE 8:**
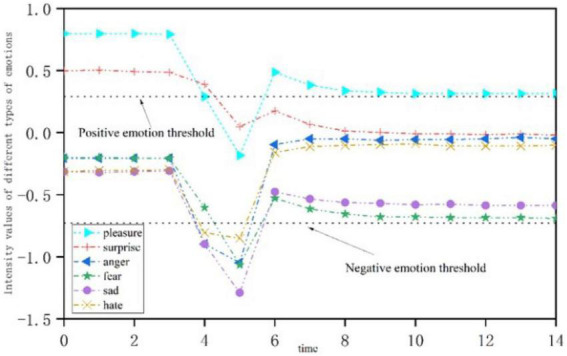
Emotional change process of open personality stimulated by a single negative stress event.

Figure 8 shows the emotional change process of open personality when it is subjected to a single negative stimulus at *t* = 3. Positive emotion appears at first, and the intensity decreases gradually, falling below the positive emotion activation threshold; meanwhile, the intensity of negative emotion increases gradually, and breaks through the threshold at *t* = 5. With the passage of time, the intensity of positive emotion gradually increased. At *t* = 6, the “pleasant” emotion is gradually higher than the threshold, while the intensity of negative emotion is gradually weakened.

**FIGURE 9:**
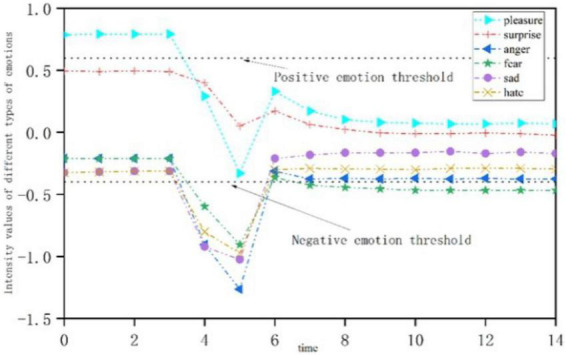
Emotional change process of neurotic personality stimulated by a single negative stress event.

Figure 9 shows the emotional change process of neurotic personality when it is subjected to a single negative stimulus at *t* = 3. The intensity of positive emotion decreases at first and then increases, but it is always below the threshold of emotional activation and is not activated. As time goes by, the intensity of most negative emotions gradually decreases, and they are higher than the threshold of negative emotions and no longer show, while “fear” emotions are gradually activated.

In negative stimulus processing, when *t* = 5, we conducted timely psychological intervention for the students who received negative stimuli, and we supported the psychological status of the students through ideological education. The results show that timely psychological intervention and ideological education support are necessary and helpful.

## Summary

Based on relevant theories of psychology, this study analyzes whether psychological crises can be warned against through continuous observation of emotions, and builds a psychological crisis warning model based on social media big data, providing innovative observation methods and ideas for the psychological crisis warning of college students. The core idea of the model is that stress events are the external cause and personality and mood changes are the internal causes.

The calculation, based on the evaluation of stress events and personality, can draw on different types of emotions and emotional threshold intensities to judge emotions. At the same time, the evaluation is based on time sequences of mood changes to judge the psychological crises that college students face by the level of risk. Combining psychological knowledge and machine learning methods, this study to improve mood on the basis of a prediction algorithm is proposed based on social media data of a psychological crisis early warning algorithm. This algorithm was created by calculating the personality, the influence of stress events, calculation of emotions, and attenuation to predict the duration and intensity, effectively avoiding the pure probability problems encountered when using machine learning algorithms. The simulation results show that the proposed algorithm can reflect the emotional changes of college students when they are subjected to stress events, and the effectiveness of the proposed algorithm is preliminarily verified.

## Data availability statement

The raw data supporting the conclusions of this article will be made available by the authors, without undue reservation.

## Author contributions

LL: conceptualization, data, formal analysis, methodology, software, supervision, validation, visualization, writing—original draft, writing—review and editing.
